# Redox-Induced *Src* Kinase and Caveolin-1 Signaling in TGF-β1-Initiated SMAD2/3 Activation and PAI-1 Expression

**DOI:** 10.1371/journal.pone.0022896

**Published:** 2011-07-28

**Authors:** Rohan Samarakoon, Subhanir S. Chitnis, Stephen P. Higgins, Craig E. Higgins, Joan C. Krepinsky, Paul J. Higgins

**Affiliations:** 1 Center for Cell Biology and Cancer Research, Albany Medical College, Albany, New York, United States of America; 2 Division of Nephrology, McMaster University, Hamilton, Ontario, Canada; Thomas Jefferson University, United States of America

## Abstract

**Background:**

Plasminogen activator inhibitor-1 (PAI-1), a major regulator of the plasmin-based pericellular proteolytic cascade, is significantly increased in human arterial plaques contributing to vessel fibrosis, arteriosclerosis and thrombosis, particularly in the context of elevated tissue TGF-β1. Identification of molecular events underlying to PAI-1 induction in response to TGF-β1 may yield novel targets for the therapy of cardiovascular disease.

**Principal Findings:**

Reactive oxygen species are generated within 5 minutes after addition of TGF-β1 to quiescent vascular smooth muscle cells (VSMCs) resulting in pp60^c-s*rc*^ activation and PAI-1 expression. TGF-β1-stimulated *Src* kinase signaling sustained the duration (but not the initiation) of SMAD3 phosphorylation in VSMC by reducing the levels of PPM1A, a recently identified C-terminal SMAD2/3 phosphatase, thereby maintaining SMAD2/3 in an active state with retention of PAI-1 transcription. The markedly increased PPM1A levels in triple *Src* kinase (*c-Src*, *Yes*, *Fyn*)-null fibroblasts are consistent with reductions in both SMAD3 phosphorylation and PAI-1 expression in response to TGF-β1 compared to wild-type cells. Activation of the Rho-ROCK pathway was mediated by *Src* kinases and required for PAI-1 induction in TGF-β1-stimulated VSMCs. Inhibition of Rho-ROCK signaling blocked the TGF-β1-mediated decrease in nuclear PPM1A content and effectively attenuated PAI-1 expression. TGF-β1-induced PAI-1 expression was undetectable in caveolin-1-null cells, correlating with the reduced Rho-GTP loading and SMAD2/3 phosphorylation evident in TGF-β1-treated caveolin-1-deficient cells relative to their wild-type counterparts. *Src* kinases, moreover, were critical upstream effectors of caveolin-1^Y14^ phosphoryation and initiation of downstream signaling.

**Conclusions:**

TGF-β1-initiated *Src*-dependent caveolin-1^Y14^ phosphorylation is a critical event in Rho-ROCK-mediated suppression of nuclear PPM1A levels maintaining, thereby, SMAD2/3-dependent transcription of the PAI-1 gene.

## Introduction

Plasminogen activator inhibitor type-1 (PAI-1, SERPINE1) is a major causative factor of arterial thrombosis and perivascular fibrosis [1]–[4] as well as a biomarker and prognostic indicator of cardiovascular disease-related death [5]. Transgenic mice that overexpress PAI-1 develop age-related vessel fibrosis and atherosclerosis while PAI-1-deficient animals are protected from experimentally-induced vascular disease [2], [6]–[9]. Since PAI-1 is involved in TGF-β1-stimulated neointima formation and lesion progression [10]–[12], clarifying the signaling network underlying TGF-β1-induced PAI-1 expression may provide novel selective targets to attenuate TGF-β1/PAI-1-associated cardiovascular pathologies.

Cooperation between non-SMAD (i.e., pp60^c-*Src*^- EGFR-ERK1/2) and SMAD signaling is required to initiate maximal TGF-β1-induced transcriptional activation of profibrotic genes such as PAI-1 and CTGF [4], [13]–[15]. SMAD2/3 phosphorylation is dependent on the ALK5 type I receptor following TGF-**β**1 ligand-receptor engagement although the maintenance of SMAD phosphorylation and, likely, SMAD function are regulated both positively and negatively by collateral mechanisms [16]. TGF-β1-stimulated Rho-ROCK activation, for example, impacts the duration (but not the initiation) of SMAD2/3 activity but the underlying molecular basis and relationship to TGF-β1 target gene transcription is unknown. TGF-β1-mediated Rho-activation, furthermore, is repressed in caveolin-1-deficient cells, perhaps due to caveolin-1/caveolae-dependent TGF-β1 receptor interactions and internalization [17]. Caveolin-1 is required for TGF-β1-mediated fibronectin expression in mesangial cells [18], however, suggesting that caveolin-1 regulation of TGF-β1 signaling may be cell type-specific.

This paper provides novel evidence that TGF-β1 stimulation of VSMC leads to a reduction in nuclear levels of PPM1A, a recently identified C-terminal SMAD2/3 phosphatase capable of attenuating TGF-β1-mediated transcriptional responses including PAI-1 expression [19]. Inhibition of Rho-ROCK signaling prior to addition of TGF-β1 rescues PPM1A expression with correlative decreases in nuclear pSMAD2/3 content implicating the Rho-ROCK pathway as an upstream negative regulator of this serine phosphatase. SMAD2/3 phosphorylation and subsequent PAI-1 induction by TGF-β1 was suppressed by genetic deficiency of caveolin-1 implicating caveolin-1 as an activator of Rho-ROCK-SMAD2/3 signaling. Src kinase activity, moreover, was critical for caveolin-1^Y14^ phosphorylation as assessed using mouse embryo fibroblasts deficient in Src, *Yes*, *Fyn* kinases (SYF^−/−/−^), by introduction of a wild-type pp60^c-*Src*^ construct in SYF^−/−/−^ cells and use of *src* kinase inhibitors. Significantly reduced SMAD3 phosphorylation and increased PPM1A expression in SYF^−/−/−^ cells, relative to wild-type fibroblasts correlated with reduced PAI-1 levels. *Src* kinase-dependent FAK phosphorylation at Y577 and Y861, moreover, is stimulated by TGF-β1 while TGF-β1-initiated FAK^Y397^ autophosphorylation was *Src*-independent. FAK is required for caveolin-1^Y14^ phosphorylation, pSMAD3 activation and PAI-1 induction. Finally, stimulation of the *Src*-FAK-caveolin-1-SMAD3 signaling axis and subsequent PAI-1 expression in response to TGF-**β**1 requires generation of reactive oxygen species (ROS) linking alterations in cellular redox state to gene reprogramming. TGF-β1 increases the production of ROS likely through several NADPH oxidases (NOXs) of which Nox4 has been linked to PAI-1 expression through mitogen-activated protein kinase phosphatase-1 inhibition [20]. While JNK and p38 appear implicated in the TGF-β1→ROS pathway of PAI-1 gene control, integration of other non-canonical SMAD-dependent events are less clear and are the subject of this study.

## Materials and Methods

### Cell Culture

Primary rat aortic VSMCs (gift of Dr. H. Singer, Albany Medical College) were cultured in DMEM/F-12 (1∶1) medium containing 10% FBS. R22 rat VSMCs (gift of Dr. P.A. Jones, USC/Norris Comprehensive Cancer Center) were grown in low glucose (1 g/l) DMEM supplemented with 10% FBS. Triple *src* family kinase (c-*src*, c-*yes*, c-*fyn*)-deficient MEFs (SYF^−/−/−^) as well as SYF^−/−/−^ cells engineered to re-express pp60^c-*src*^ (also from Dr. H. Singer), caveolin-1-null MEFs and their wild-type counterparts (provided by Dr. P.J. McKeown-Longo, Albany Medical College) and FAK-deficient MEFs and corresponding wild-type cells (gift of Dr. J. Zhao, Albany Medical College) were propagated in DMEM containing 10% FBS. Conditions for serum-deprivation and TGF-β1 stimulation for each cell type is described in the text as is pretreatment with SU6656 (*src* family kinase inhibitor), Y-27632 (p160ROCK inhibitor), SIS3 (SMAD3 inhibitor) (all from Calbiochem). Inhibitors of free radical generation, N-acetyl cysteine (NAC) and diphenyleneiodonium chloride (DPI), were from Sigma-Aldrich.

### Western Blotting

VSMCs and MEFs were disrupted in 4% SDS/PBS for 10 minutes, lysates vortexed briefly, boiled for 5 minutes then centrifuged at 14,000 rpm for 15 minutes. Aliquots (30 µg cellular protein) were electrophoretically-separated, transferred to nitrocellulose, membranes blocked in 5% milk in 0.05% Triton-X 100/PBS, incubated overnight with specific antibodies to rat PAI-1 (American Diagnostica), EGFR, pEGFR^Y845^, pSMAD2^Ser465/467^, SMAD2/3, pSMAD3^Ser423/425^, pp60^c-*src*^-pY416 (Cell Signaling); pERK1/2, ERK2, pSMAD 2/3, FAK, RhoA, TGF-βRI (Santa Cruz Biotechnology), phosphotyrosine (4G10, Upstate Biotechnology), caveolin-1, phospho-caveolin-1^Y14^ (BD Bioscience), pFAK^Y397^, pFAK^Y577^, pFAK^Y861^ (Biosource), and human PAI-1 (#9163) in blocking buffer and washed three times in 0.05% Triton-X 100/PBS prior to incubation with secondary antibodies. Immunoreactive proteins were visualized with ECL reagent and quantitated by densitometry. Stripped membranes were reprobed with antibodies to actin, EGFR, caveolin-1, RhoA, ERK2, pp60^c-*src*^, SMAD2 or SMAD2/3 to confirm protein loading levels. Statistical analysis of quantitative data from scanned blots was done by t-test.

### Immunohistochemistry and Immunocytochemistry

Tissue sections of human carotid artery plaques (gift of Dr. M. Lennartz, Albany Medical College) were de-paraffinized in 3 changes of xylene (5 mins each), placed in 2 changes of 100% ethanol (3 mins each), hydrated in progressively-diluted ethanol 95% (3 mins), 70% (3 mins) and 50% (3 mins) and rinsed in distilled water. Slides were immersed in sodium citrate buffer (10 mM sodium citrate, 0.05% Tween 20, pH 6.0), heated at 95–100°C for 15 minutes then cooled to room temperature. Following several PBS washes, sections were blocked with 10% normal goat serum for 1 hour, incubated in primary antibodies to PAI-1 (#9163) and α-smooth muscle actin (10 µg/ml; Sigma Aldrich), diluted in 1% BSA/PBS, washed in PBS (3 times, 5 mins each), then incubated in appropriate secondary antibodies (Molecular probes; Alexa series) diluted in 1% BSA in PBS for 1 hour. After final washing in PBS (3×5 mins each), sections were mounted with ProLong antifade-gold+DAPI. For immunocytochemistry, serum-deprived semi-confluent MEFs and VSMCs were stimulated with TGF-β1 (2 hours) and processed for immunofluorescence as described previously [13], [15]. Briefly, cells were fixed in 3% paraformaldehyde, permeabilized in 0.25% Triton X-100, blocked in goat serum then overlayed with antibodies to caveolin-1 or pcaveolin-1 (1∶200) for a 1 hour incubation at 37°C. Following 3 PBS washes, cells were incubated in Alexa 488-labeled secondary antibodies prior to final rinsing and mounting as detailed above.

### Rho GTPase Assay

PBS-washed cells were extracted in 25 mM HEPES, pH 7.5, 150 mM NaCl, 1 mM EDTA, 10% glycerol containing leupeptin and 1 mM sodium orthovanadate) by constant agitation for 15 minutes at 4°C. Clarified lystates (600 µg protein) were incubated with Rhotekin RBD-agarose beads for 45 minutes at 4°C. Active (i.e., Rhotekin-bound) Rho and total Rho levels (GTP-Rho+GDP-Rho) were determined by western blotting with RhoA antibodies.

### Transient Transfection of siRNA or Dominant-Negative (DN) Constructs

Semi-confluent (70%) primary VSMC cultures were washed in PBS prior to addition of siRNA constructs to GFP (control), SMAD3, caveolin-1 or PPM1A (Dharmacon; final concentration 1 mM), in Accell siRNA delivery medium (1 ml) for 72–96 hours. Following a brief incubation in serum-free DMEM, VSMCs were stimulated with TGF-β1 for 4 hrs prior to harvesting for extraction. Subconfluent 35-mm cultures of R22 cells were transfected with DN-pp60^c-*src*^, DN-RhoA^N17^ or control GFP expression constructs as described [13]–[15]. Following transfection, cells were serum-deprived for 2 days prior to TGF-β1 stimulation. Transfection efficiency was 50–70% (assessed by GFP fluorescence microscopy).

### Generation of Stable Cell Lines

Wild-type caveolin-1 (Cav-1^WT^) pLHCX retroviral expression constructs [18] were transfected into sub-confluent caveolin-1^−/−^ MEFs using Lipofectimine (1∶3 DNA/lipid ratio) in DMEM for 6 hours. Following overnight recovery in DMEM/10% FBS, transfectants were selected in hygromycin (200–350 µg/ml) for 5–7 days.

### Immunoprecipitation

Cells were disrupted for 30 min (in cold 50 mM HEPES, pH 7.5, 1% Triton X-100, 1% NP-40, 0.5% deoxycholate, 150 mM NaCl, 50 mM NaF, 1 mM Na-orthovanadate, 0.1% SDS, protease cocktail inhibitor) and extracts clarified at 14,000 g for 15 min. Lysate protein (500 µg) from control and TGF-β1-treated cells were incubated with antibodies to RhoA (2 µg, RhoA; Santa Cruz Biotechnology) for 2 h in a total volume of 500 µl. Immune complexes were collected with Protein A/G Plus-agarose, washed three times with lysis buffer without SDS and boiled in sample buffer.

### Reactive Oxygen Species (ROS) Assay

The carboxy derivative of fluorescein, 2′,7′-dichlorofluorescein (carboxy-H2DCFDA) (Molecular probes; C400) was used to determine ROS generation in response to TGF-β1 according to manufacturer's recommendations. Briefly, cells were stimulated with TGF-β1 for the times indicated, medium removed and cells incubated with 5 µM DCFDA in PBS for 15 minutes prior to scrape harvest. Equivalent number of cells were used to assess baseline fluorescence (unstimulated) and response to TGF-β1 stimulation with a multi-detection microplate reader (Synergy HT; Bio-Tek) at an excitation wavelength of 495 nm.

## Results

**Figure 1:**
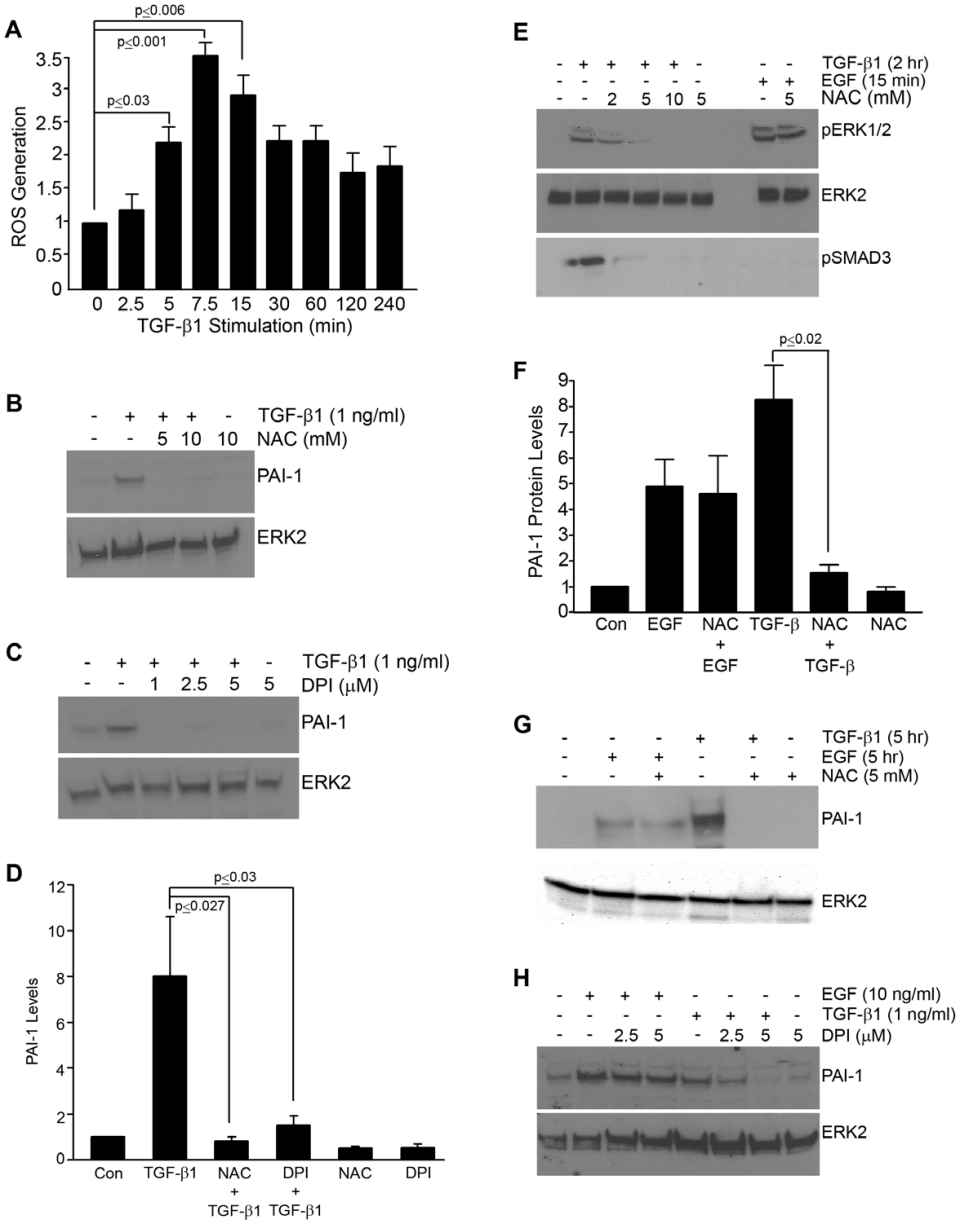
PAI-1 induction in response to TGF-β1 involves reactive oxygen species (ROS). DCF fluorescence measurements (as described in Methods) were used to determine ROS generation (per equivalent number of cells) and expressed relative to unstimulated cultures (set as a.u. = 1). ROS levels increase within 5 minutes after addition of TGF-β1 (1 ng/ml) to serum-deprived quiescent VSMCs (**A**). ROS generation appears to be important in TGF-β1-stimulated PAI-1 expression since PAI-1 induction is effectively suppressed by even low concentrations of the established inhibitors of free radical generation NAC (**B**) and DPI (**C**). NAC pretreatment also attenuates (at 2 mM) and completely eliminates (at concentrations ≥5 mM) TGF-β1-dependent ERK1/2 and SMAD2/3 phosphorylation but has no effect of EGF-stimulated ERK1/2 activation (**E**). Both NAC (**B,F,G**) and DPI (**C,H**) pretreatment (30 mins) served to assess the role of ROS in TGF-β1- and EGF-mediated PAI-1 induction. ERK2 provided a loading control. Data plots (**A,D,F**) represent the mean ± S.D. of three independent experiments; statistical significance among the indicated groups was calculated by t-test.

ROS are rapidly generated (within 5 minutes) in response to TGF-β1 (**Figure 1A**). Pretreatment of VSMCs with NAC (a glutathione precursor) (**Figure 1B**) or DPI (which inhibits nitric oxide synthetase and NADPH oxidase) (**Figure 1C**) effectively suppressed PAI-1 induction by TGF-β1 (summarized in **Figure 1D**) and reduced TGF-β1-mediated ERK1/2 as well as SMAD2/3 activation (**Figure 1E**). NAC, however, did not affect EGF-stimulated ERK1/2 phosphorylation (**Figure 1E**) and, in contrast to requirements for TGF-β1 induction, neither NAC (**Figure 1F,G**) or DPI (**Figure 1H**) blocked EGF-stimulated PAI-1 expression. The involvement of ROS in PAI-1 gene control is clearly stimulus-dependent.

**Figure 2:**
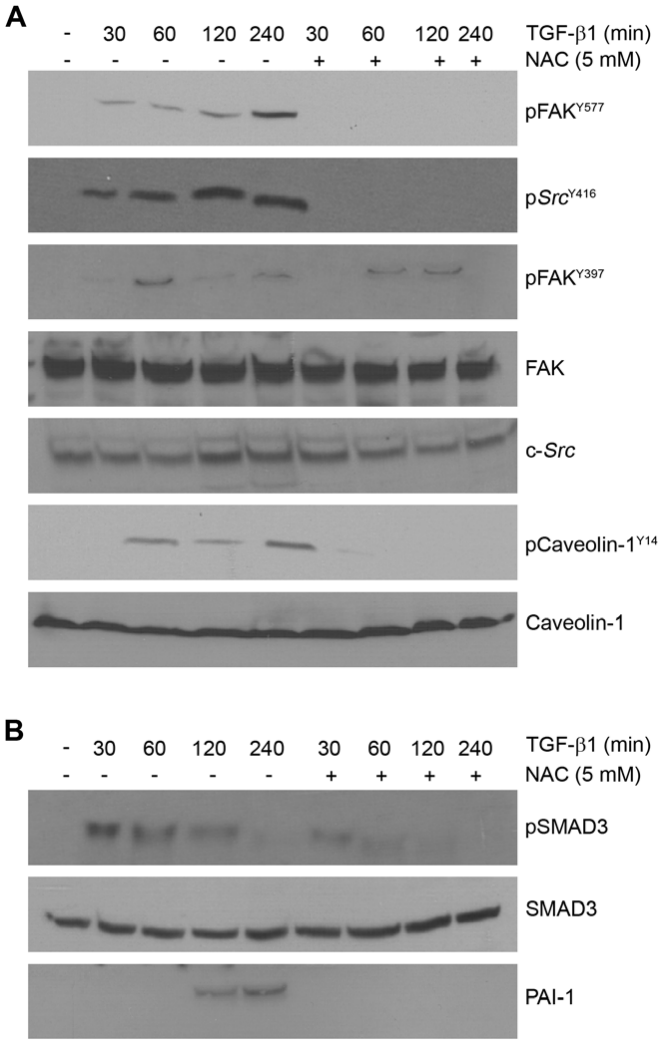
Inhibition of ROS generation attenuates TGF-β1 signaling in VSMC. Quiescent VSMCs were stimulated with TGF-β1 (1 ng/ml) for the times indicated with or without NAC (5 mM) pretreatment for 1 hour. Increases in p*Src* ^Y416^, pFAK^Y577^ and pCaveolin^Y14^ (targets of c-*Src* kinases) in response to TGF-β1 is completely inhibited by NAC, suggesting an upstream role for ROS generation in activation of Src/FAK/caveolin-1 signaling pathways (**A**). FAK^Y397^ phosphorylation by TGF-β1 (at least within the time frame of 2 hours) is relatively unaffected by NAC blockade of ROS generation. Total levels of *c-Src*, FAK and caveolin-1 are largely unchanged over the time course of TGF-β1 exposure serving as loading controls (**A**). To assess the role of ROS generation in SMAD3 activation, TGF-β1-stimulated SMAD3 phosphorylation over time was compared to an identical window with NAC pretreatment. Blots were probed with antibodies to determine both pSMAD3 and total SMAD3 levels (**B**).

Since changes in redox state by TGF-β1 affects both the canonical SMAD and non-canonical pathways (e.g., **Figure 1E**), it was important to clarify the impact on downstream TGF-β1 effectors (e.g., *src*, EGFR, FAK, caveolin-1, SMADs). NAC effectively suppressed TGF-β1-induced c-*Src* ^Y416^ as well as FAK^Y577^ (a target of activated *c-Src* kinases) phosphorylation (**Figure 2A**), positioning ROS upstream of c-*Src* -and FAK- mediated signaling. While TGF-β1-stimulated caveolin-1^Y14^ phosphorylation is also NAC sensitive, FAK^Y397^ autophosphorylation is only marginally affected by NAC pretreatment suggesting the participation of non-ROS-dependent mechanisms in FAK auto-activation (**Figure 2A**). Time-course assessments indicated, moreover, that NAC preincubation suppressed both the amplitude and duration of SMAD3 phosphorylation as well as the inhibition of PAI-1 induction (cf., **Figures 1E** **,** **2B**). Consistent with suppression of SMAD3 phosphorylation, PAI-1 induction by TGF-β1 is also effectively attentuated by NAC preincubation (**Figure 1D** **,** **2B**).

**Figure 3:**
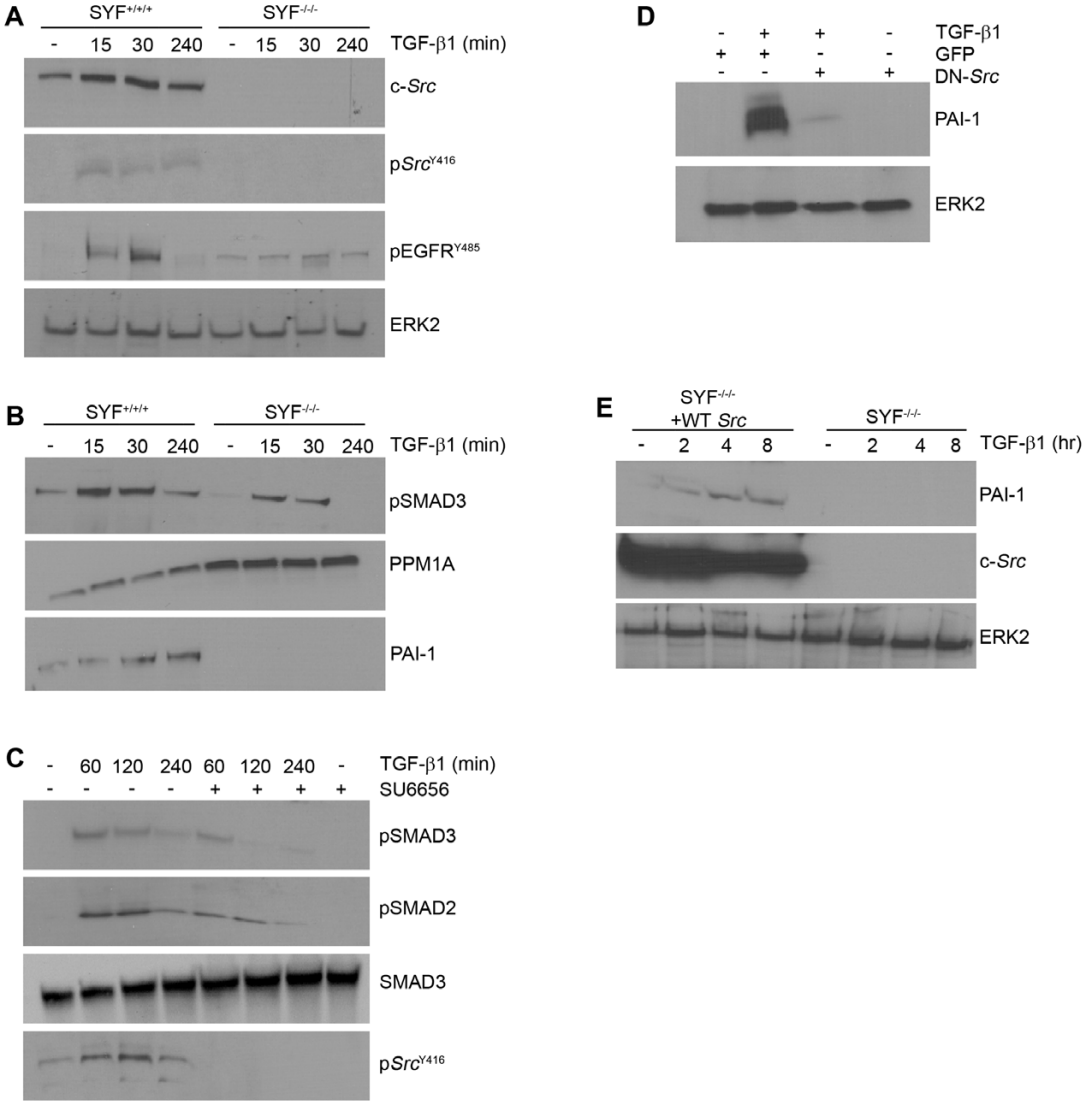
Downstream signaling events initiated by TGF-β1-activated S*rc* kinase. SYF^+/+/+^ and SYF^−/−/−^ fibroblasts were serum-deprived for 1 day prior to stimulation with TGF-β1 (0.1 ng/ml) for the times indicated and lysates subject to western analysis. *Src* activation (assessed using phospho- *Src* ^Y416^ antibodies) and increased EGFR phosphorylation at the *Src* kinase target Y845 site, are both evident in TGF-β1-stimulated wild-type (SYF^+/+/+^) MEFs but not *Src*, *Fyn*, *Yes* triple-null (SYF^−/−/−^) cells (**A**). The level (at 15 and 30 minutes) and maintenance (at 4 hrs) of SMAD3 phosphorylation is significantly reduced in SYF^−/−/−^ fibroblasts compared to their wild-type counterparts (**B**). In contrast to the typical time course-dependency of PAI-1 induction in response to TGF-β1 in SYF^+/+/+^ cells, PAI-1 was not detectable in *Src*-deficient MEFs regardless of the duration of TGF-β1 exposure. The absence of PAI-1 expression and attenuated SMAD3 phosphorylation reflected increased PPM1A levels in SYF^−/−/−^ as compared to SYF^+/+/+^ fibroblasts (**B**). Pretreatment of VSMCs with the *Src* kinase inhibitor SU6656 (2 µM) blocked the long-term maintenance (but not the initiation) of SMAD2/3 phosphorylation in response to TGF-β1 while total SMAD levels remain unchanged (**C**). *Src* ^Y416^ phosphorylation by TGF-β1 was completely eliminated by SU6656 confirming the effectiveness of this inhibitor (**C**). Transient transfection of VSMCs with a dominant-negative pp60^c-*src*^ (DN-*Src*) expression construct (or a GFP control vector) 72 hours prior to incubation with TGF-β1 for 6 hours was followed by western analysis for PAI-1. TGF-β1-stimulated PAI-1 induction was effectively suppressed by the DN-*Src* but not the GFP construct (**D**). SYF^−/−/−^ cells genetically-engineered to express wild-type pp60^c-*src*^ (SYF^−/−/−^+WT *Src*) rescued PAI-1 inducibility in response to TGF-β1 (**E**). ERK2 (**A,D,E**) and SMAD3 (**C**) serve as a loading controls.

Given the importance of *Src* kinases as downstream effectors of ROS- sensitive pathways [21], the *Src*-dependency of TGF-β1-initiated signaling was further assessed using *Src*, *Yes*, *Fyn* triple-null (SYF^−/−/−^) and wild-type (SYF^+/+/+^) MEFs. c-*Src* protein as well as c-*Src* ^Y416^ phosphorylation was evident, as expected, in TGF-β1-stimulated wild-type but not SYF-null cells (**Figure 3A**). EGFR activation in response to TGF-β1, moreover, is significantly diminished in SYF^+/+/+^ compared to SYF^−/−/−^ fibroblasts consistent with involvement of Src kinases in TGF-β1-mediated EGFR transactivation in VSMCs [13]–[15]. SMAD3 phosphorylation (both extent and duration) is also significantly reduced in SYF^−/−/−^ cells compared to their wild-type counterparts over the time course of TGF-β1-stimulation and PAI-1 induction is completely eliminated in *Src* kinase-deficient MEFs (**Figure 3B**). This is in keeping with the higher levels of PPM1A evident in SYF^−/−/−^ relative to wild-type fibroblasts. VSMC pretreatment with the *src* kinase-specific inhibitor SU6656, as expected, prevented the TGF-β1-dependent increase in c-*Src* ^Y416^ phosphorylation (**Figure 3C**). SU6656, however, did not impact TGF-β1-initiated SMAD2/3 activation at early time points (e.g., 1 hour) but completely eliminated later-stage (e.g., 4 hrs) SMAD2/3 phosphorylation (**Figure 3C**). Transient transfection of VSMCs with a dominant-negative c-*Src* construct, furthermore, effectively inhibited PAI-1 expression upon TGF-β1 addition (**Figure 3D**). Stable reconstitution of wild-type pp60^c-*src*^ in SYF^−/−/−^ cells (SYF^−/−/+WT-*Src*^) was sufficient to “rescue” TGF-β1-mediated PAI-1 inducibility (**Figure 3E**) confirming participation of pp60^c-*src*^ in PAI-1 gene control.

**Figure 4:**
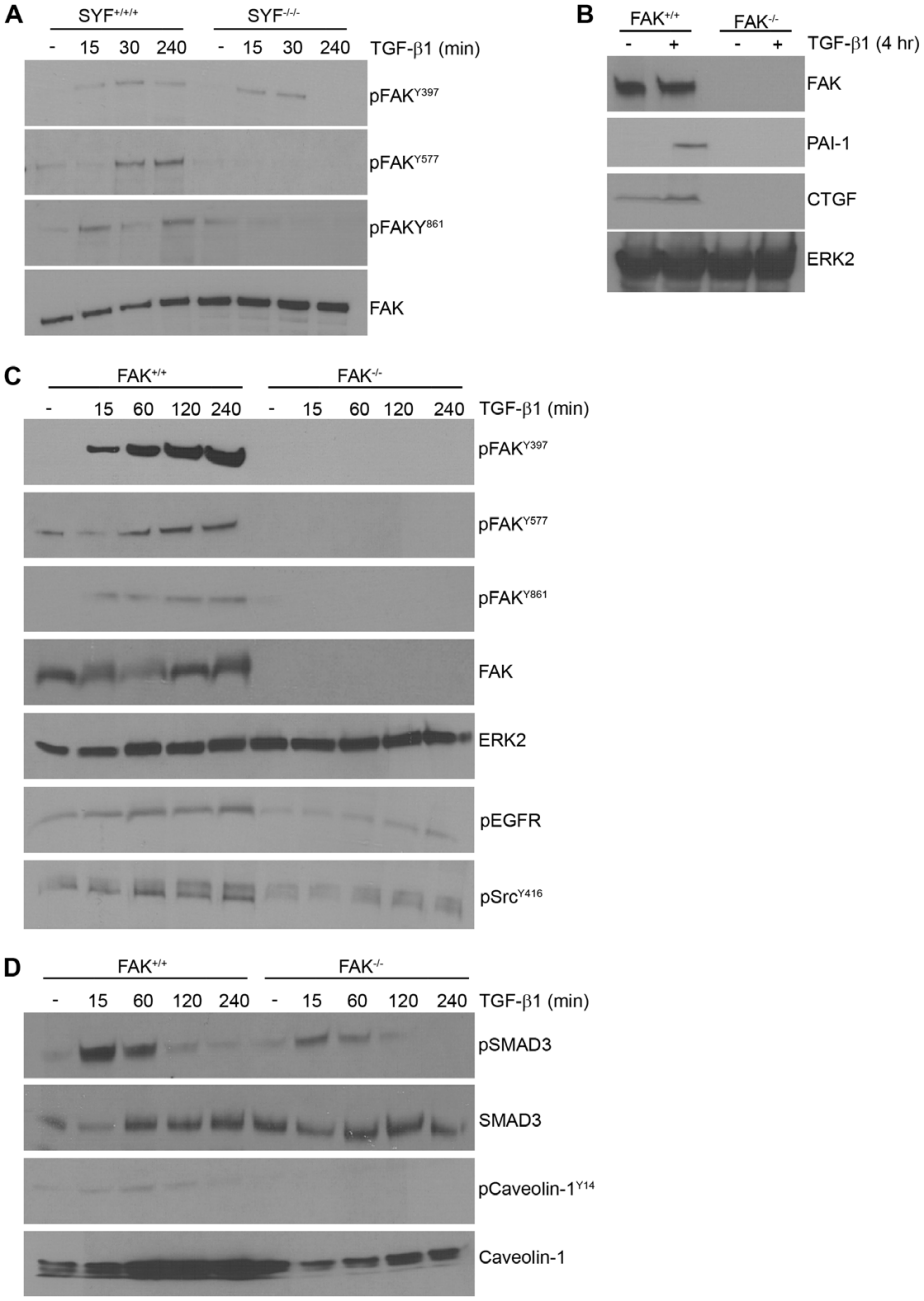
FAK is a downstream target of *Src* kinases and is required for PAI-1 induction by TGF-β1. MEFs were serum-deprived for 1 day prior to addition of TGF-β1 (0.1 ng/ml). TGF-β1 stimulates FAK phosphorylation at the Y577 and Y861 sites in SYF^+/+/+^ but not SYF^−/−/−^cells consistent with an upstream role of *Src* kinases in FAK activation. TGF-β1-induced FAK^Y397^ autophosphorylation, in contrast, is unaffected by genetic ablation of *src* family kinases (**A**). To assess the role of FAK in TGF-β1-induced PAI-1 and CTGF expression, serum-deprived FAK^+/+^ and FAK^−/−^ MEFs were stimulated with TGF-β1 and blots probed with antibodies to PAI-1 and CTGF (**B**). TGF-β1 stimulates FAK phosphorylation at Y397, Y561 and Y861 only in wild-type but not, as anticipated, in FAK-null fibroblasts (**C**) providing antibody specificity controls for panels **A–C**. TGF-β1-stimulated c-*Src* and EGFR activation is significantly attenuated in FAK^−/−^ cells relative to FAK^+/+^ MEFs (**C**). SMAD3 C-terminal phosphorylation in response to TGF-β1 is reduced in FAK^−/−^ as compared to FAK^+/+^ cells; total SMAD2/3 levels were unchanged regardless of FAK genetic status (**D**). Western analysis was used to evaluate the effect of FAK genetic status (FAK^−/−^ vs. FAK^+/+^) on TGF-β1-induced caveolin-1^Y14^ phosphorylation (**D**). Consistent with previous observations [40], total caveolin-1 is lower in FAK^−/−^ MEFs compared to wild-type cultures (**D**). Assessment of total FAK (**A,B**), ERK2 (**B,C**) and SMAD3 (**D**) provided loading controls.

Since TGF-β1 stimulates FAK tyrosine phosphorylation (at Y397, Y577 and Y861), it was necessary to assess whether *Src* kinases are upstream regulators of this response reminiscent of *Src*-FAK involvement in adhesion-based signaling (e.g., [22]–[25]). pFAK^Y397^ levels were similar in SYF^−/−/−^ cells and wild-type fibroblasts suggesting that TGF-β1-initiated FAK autophosphorylation is largely *Src*-independent (**Figure 4A**). However, TGF-β1-initiated FAK^Y577 and Y861^ phosphorylations are not evident in SYF^−/−/−^ fibroblasts compared to wild-type MEFs confirming a role for *Src* kinases in FAK activation in response to TGF-β1. FAK is critical, moreover, for both TGF-β1-induced PAI-1 and CTGF expression as neither are detectable in FAK-null MEFs (**Figure 4B**). TGF-β1-induced FAK^Y397,Y577,Y861^ phosphorylation is also evident in FAK^+/+^ MEFs (similar to VSMCs) but not in their null counterparts as anticipated (**Figure 4C**). FAK appears critical, moreover, for optimal c-*Src* kinase activation by TGF-β1 since *Src* ^Y416^ phosphorylation is dramatically decreased in FAK^−/−^ fibroblasts compared to wild-type cells. FAK^−/−^ MEFs, furthermore, do not increase EGFR^Y845^ phosphorylation in response to TGF-β1 consistent with an upstream role of c-*Src* and FAK in TGF-β1-initiated EGFR transactivation (**Figure 4C**). FAK deficiency also impacted other TGF-β1 signal intermediates as well. SMAD3 activation in TGF-β1-treated FAK^−/−^ cells is substantially reduced compared to wild-type MEFs despite equivalent SMAD3 protein levels regardless of genetic background (**Figure 4D**).

**Figure 5:**
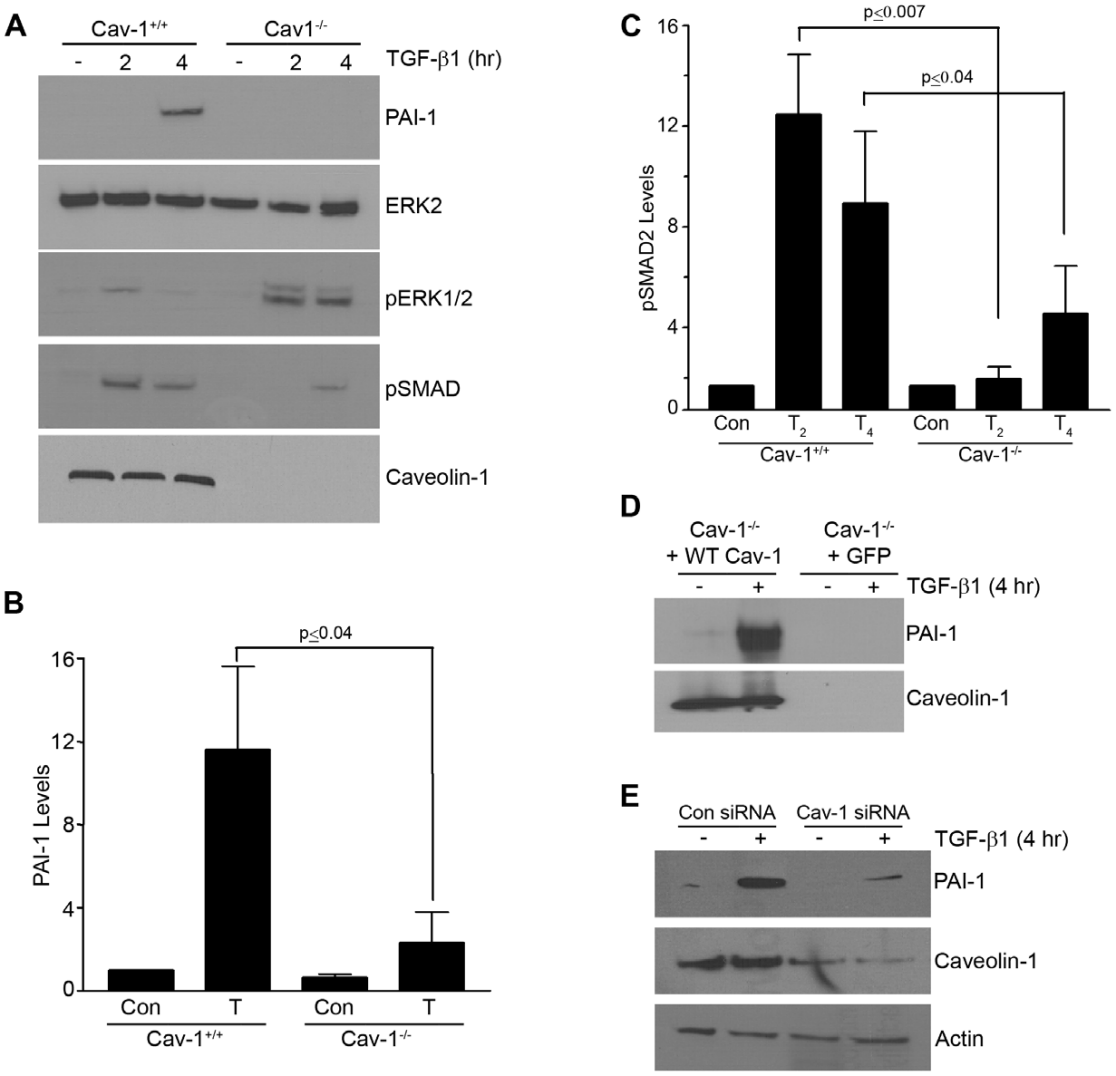
Caveolin-1 is required for TGF-β1-induced PAI-1 expression. Serum-deprived (1 day) caveolin-1^+/+^ and caveolin-1^−/−^ MEFs were stimulated with TGF-β1 (0.1 ng/ml) for 2 or 4 hours and blots probed with antibodies to PAI-1, pSMAD2 and pERK1/2. PAI-1 induction is apparent in wild-type but not in caveolin-1-deficient cells (**A,B**). TGF-β1-induced SMAD2 phosphorylation is decreased while ERK1/2 activation is increased in caveolin-1-null compared to wild-type fibroblasts at comparable time points (**A,C**). Exposure to TGF-β1 (T) was for 4 hours in (**B**) and for 2 or 4 hours in (**C**). Introduction of a wild-type caveolin-1 construct (+WT Cav-1) in caveolin-1-null cells rescues TGF-β1 inducibility of PAI-1 unlike caveolin-1^−/−^ MEFs expressing GFP (+GFP) (**D**). VSMCs were transfected with control or caveolin-1 siRNA constructs and, after a brief period of serum deprivation, stimulated with TGF-β1 for 4 hours. Cellular lysates were separated by electrophoresis and blots probed with antibodies to PAI-1, caveolin-1 and actin (as a loading control) (**E**). Histograms (**B,C**) depict the mean ± S.D. of three independent experiments.

The TGF-β1-dependent increase in caveolin-1^Y14^ phosphorylation was similarly attenuated by FAK deficiency with consequences on TGF-β1 signaling (**Figure 4D**) since caveolin-1^−/−^ cells have a significantly decreased PAI-1 inductive response compared to Cav-1^+/+^ MEFs (**Figure 5A,B**). The level and time course of TGF-β1-stimulated SMAD2/3 activation were both decreased in caveolin-1^−/−^ fibroblasts (as was the case in NAC- or SU6656-treated cells as well as in SYF^−/−/−^ or FAK^−/−^ cells), while ERK1/2 phosphorylation, in contrast, is increased (**Figure 5A,C**). Stable re-introduction of a wild-type caveolin-1 construct (WT cav-1) in caveolin-1^−/−^ cells rescued PAI-1 expression (**Figure 5D**) confirming a role of caveolin-1 in TGF-β1 signaling in fibroblasts. Consistent with these findings, transient siRNA-mediated knockdown of caveolin-1 expression effectively suppressed PAI-1 induction in TGF-β1-stimulated VSMCs compared to control siRNA-transfected cultures (**Figure 5E**).

**Figure 6:**
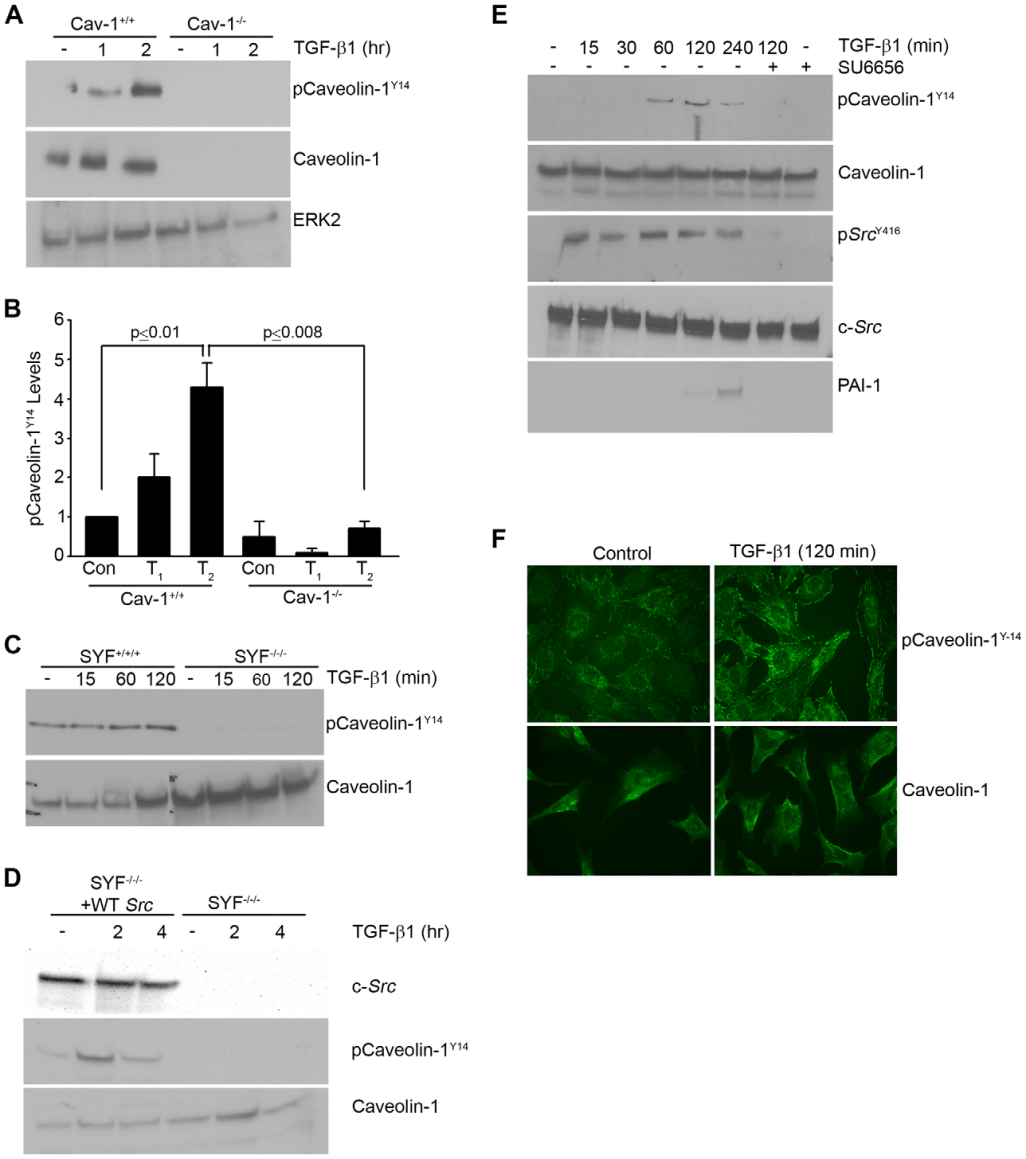
c-*Src* is an upstream regulator of caveolin-1^Y14^ phosphorylation. MEFs were serum-deprived for 1 day prior to incubation with TGF-β1 (0.1 ng/ml) for the times indicated. Western analysis indicated that TGF-β1 stimulated caveolin-1 phosphorylation at the Y14 c-*Src* kinase target site in caveolin-1^+/+^ fibroblasts but not, as expected, in caveolin-1^−/−^ cells (**A,B**). Caveolin^Y14^ phosphorylation is similarly evident extracts of SYF^+/+/+^ but not in SYF^−/−/−^ MEFs (**C**). Stable expression of a pp60^c-*src*^ construct (+WT *Src*) in SYF^−/−/−^ fibroblasts is sufficient to rescue caveolin^Y14^ phosphorylation in response to TGF-β1 (but not in empty vector expressing SYF^−/−/−^ cells) despite comparable caveolin-1 expression in both cell types (**D**). Pretreatment of serum-deprived VSMC with the *Src* kinase inhibitor SU6656 (2 µM) prior to addition of TGF-β1 (1 ng/ml) eliminated TGF-β1-induced *Src* ^Y416^ activation, caveolin^Y14^ phosphorylation and PAI-1 expression (**E**). Total ERK2 (**A,B**), caveolin-1 (**C,D,E**) and c-*Src* (**E**) were approximately constant under all culture conditions providing internal loading controls. Data plotted in (**B**) represent the mean ± S.D. of three independent experiments. To assess potential growth factor-associated changes in caveolin-1 localization, subconfluent serum-deprived MEFs were stimulated with TGF-β1 (0.1 ng/ml) for 2 hrs and the distribution of phospho-caveolin-1^Y14^ and total caveolin-1 assessed by immunocytochemistry; control cells remained untreated (**F**).

The functional state of caveolin-1 is subject to modulation by Y14 phosphorylation, by subcellular location (e.g., caveolae, focal contacts or lipid rafts), or by expression levels [26]–[29]. TGF-β1-induced caveolin-1^Y14^ phosphorylation is evident within 1–2 hrs in wild-type MEFs but not, as anticipated, in caveolin-1-null cells (**Figure 6A,B**). Since caveolin-1^Y14^ is a substrate of the Abelson (Abl) and *Src* kinases, albeit under different restrictions [26], the role of *Src* in caveolin Y14 site targeting in the context of TGF-β1 stimulation was assessed. Caveolin-1^Y14^ phosphorylation in response to TGF-β1 was undetectable in SYF^−/−/−^ cells (**Figure 6C**). Stable re-expression of pp60^c-*src*^ in SYF^−/−/−^ cells rescued caveolin-1^Y14^ phosphorylation as well as PAI-1 induction (**Figure 3B** **,** **6D**). To assess if caveolin-1^Y14^ phosphorylation in VSMCs is similarly mediated by *Src* kinases, quiescent cultures were pretreated with SU6656 (2 µM) prior to addition of TGF-β1. TGF-β1-stimulated caveolin-1^Y14^ phosphorylation, c-*Src* ^Y416^ site activation and subsequent PAI-1 expression were completely eliminated by SU6656 (**Figure 6E**). Caveolin-1 trafficking also appears to be phosphorylation state-dependent as phospho-caveolin-1 redistributed to focal adhesion-like peripheral structures within 2 hrs of TGF-β1 stimulation (**Figure 6F**) indicating that changes in subcellular distribution occur within the real time of PAI-1 induction.

**Figure 7:**
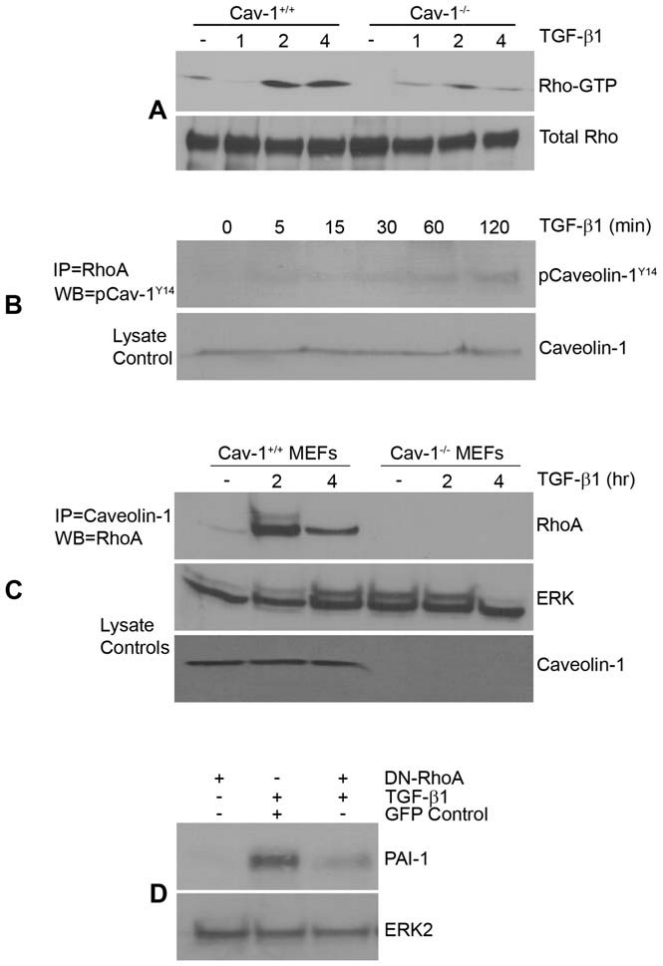
RhoA both interacts with caveolin-1^Y14^ in response to TGF-β1 and required for PAI-1 induction. A Rho-GTPase assay (as described in Methods) was used to assess relative RhoA activation by TGF-β1 in fibroblasts. RhoA-GTP loading increased within 2–4 hours of TGF-β1 addition (0.1 ng/ml) to 1-day serum-deprived wild-type MEFs. In contrast, the level and duration of RhoA activation during this 4 hour window is markedly reduced in caveolin-1-null fibroblasts compared to caveolin-1^+/+^ cells (**A**). Immunoprecipitation (IP) of RhoA followed by phospho-caveolin-1^Y14^ western analysis disclosed a time-dependent association between phospho-caveolin-1^Y14^ and endogenous RhoA in response to TGF-β1 while total levels of caveolin-1 remain unchanged (**B**). IP of caveolin-1 followed by western blotting for RhoA similarly confirmed increased interaction between both proteins in wild-type (WT) MEFs upon a 2 to 4 hr stimulation with TGF-β1 but not in caveolin-null cells (**C**). Transfection of a dominant-negative RhoA construct prior to addition of TGF-β1 effectively inhibited PAI-1 expression while introduction of a GFP control vector was without effect (**D**) indicating that RhoA is required for TGF-β1-induced PAI-1 expression.

**Figure 8:**
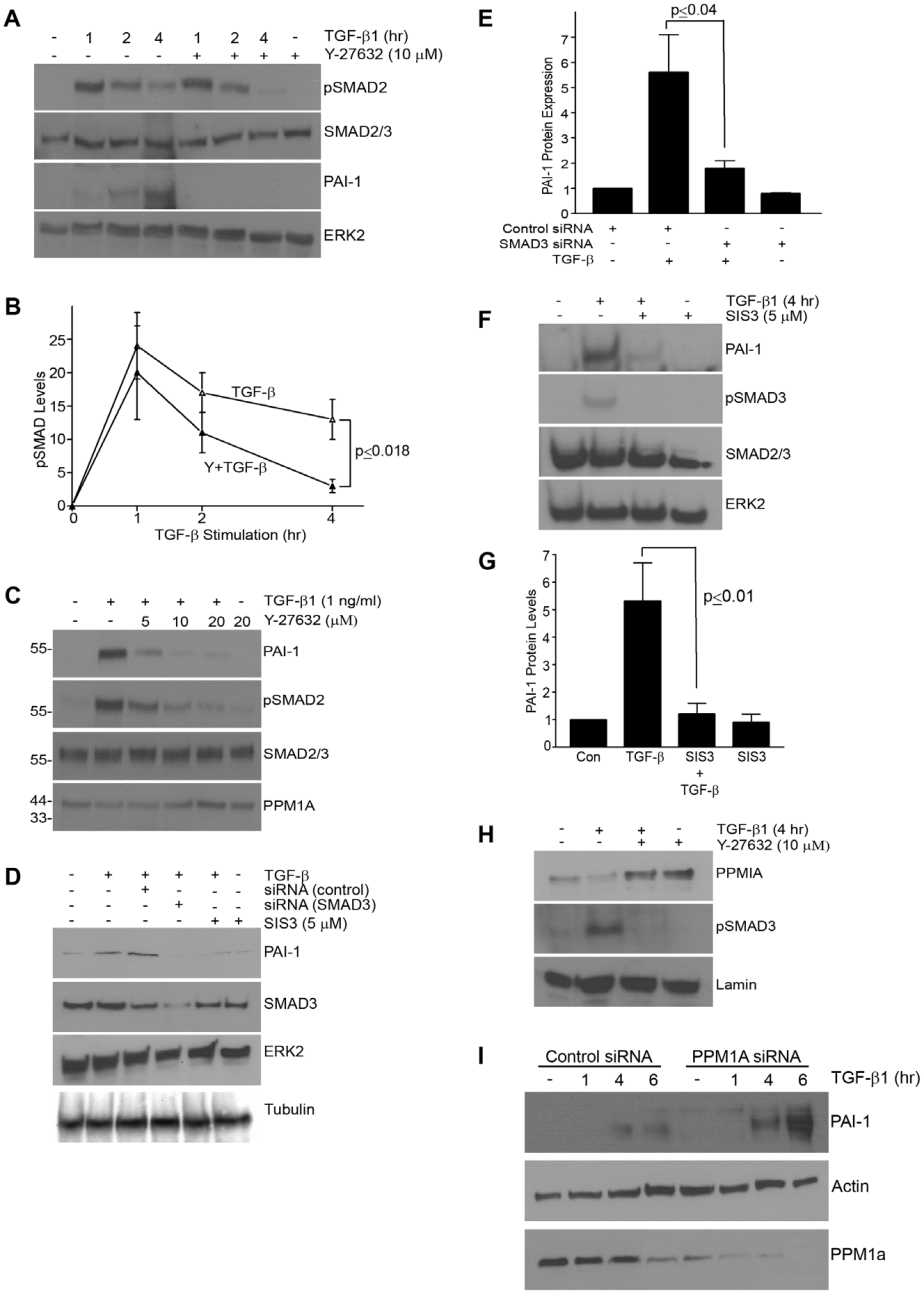
Rho-ROCK pathway regulates nuclear levels of PPM1A and maintains SMAD3 activation. VSMCs maintained under serum-deprived conditions for 1 day were TGF-β1-stimulated (1 ng/ml) with or without the ROCK inhibitor, Y-27632 (10 µm) and cellular lysates probed for pSMAD2, total SMAD2/3, PAI-1 and ERK2 (**A**). Late-stage (4 hour) pSMAD2 levels were markedly attenuated and PAI-1 expression completely inhibited by ROCK blockade (**A**). TGF-β1-induced SMAD phosphorylation at the late time points (4 hours) is significantly reduced by inhibition of ROCK signaling. Serum-deprived VSMCs were pretreated for 30 minutes with Y-27632 (at indicated concentrations) prior to exposure to TGF-β1 for 4 hours. Cell lysates were probed for PAI-1, SMAD2/3, pSMAD2/3 and PPM1A (**C**). PAI-1 expression in response to TGF-β1 was completely blocked by Y-27632 pre-exposure (10 µM final concentration) despite the initial increase in SMAD2 phosphorylation in Y-27632-treated cells. Concentrations of Y-27632 that effectively inhibit PAI-1 induction and suppress SMAD2 phosphorylation also increase PPMIA levels (**C**). Transient knock-down of SMAD3 with siRNA constructs (as detailed in Methods) (**D,E**) or pre-incubation with the small molecule inhibitor of SMAD3 phosphorylation SIS3 (5 µM) [41] (**D,F,G**) eliminates TGF-β1-induced PAI-1 expression in VSMCs (**D,E**) and MEFs (**F,G**). Cell fractionation studies confirmed that nuclear accumulation of pSMAD3 in response to TGF-β1 is blocked while nuclear PPM1A content increased upon pre-incubation with Y-27632 (**H**). TGF-β1 stimulation for 4 hours actually reduced nuclear PPM1A levels, which was restored by Y-27632 pretreatment (**H**). siRNA-mediated PPM1A knockdown in VSMCs resulted in a significantly increased TGF-β1-induced PAI-1 response compared to cells transfected with control siRNA constructs (**I**). ERK2 (**A,B,D–G**), SMAD2/3 (**A,C,F**), tubulin (**D**), lamin (**H**) and actin (**I**) provide loading controls. Data plotted in (**B,E,G**) is the mean ± S.D. of three independent experiments.

To investigate downstream targets of caveolin-1 in transducing TGF-β1 signals, focus centered on RhoA as TGF-β1 stimulates Rho GTP loading (**Figure 7A**). Caveolin-1 interacts with RhoA in response to TGF-β1 (**Figure 7B,C**) and active RhoA (2–4 hrs post TGF-β1 stimulation) is markedly reduced in caveolin-1^−/−^ compared to wild-type fibroblasts despite equivalent RhoA levels (**Figure 7A**). Transient expression of a DN-RhoA construct or pre-incubation with the ROCK inhibitor Y-27632 eliminated PAI-1 induction by TGF-β1 establishing the signaling relevance of an intact RhoA-ROCK pathway in PAI-1 gene control (**Figures 7D** **,** **8A**). Time-course and dose-response assessments indicated, furthermore, that ROCK inhibition only marginally affected TGF-β1-induced pSMAD2/3 levels at one hour but completely blocked SMAD2/3 phosphorylation and nuclear accumulation at 4 hours (**Figure 8A–C**) suggesting that the Rho-ROCK pathway impacts not the initiation but the maintenance of SMAD2/3 phosphorylation. SMAD3 is, in fact, a critical downstream effector of TGF-β1-dependent PAI-1 expression as SMAD3 knockdown (**Figure 8D,E**) or pre-treatment with SIS3 (a selective inhibitor of SMAD3 phosphorylation) (**Figure 8D,E**) completely suppressed PAI-1 induction in VSMCs (**Figure 8D** **; not shown**) as well as in MEFs (**Figure 8F,G**). Nuclear levels of pSMAD3 increase over the 4 hour time course response to TGF-β1 stimulation as expected; Y-27632 preincubation virtually eliminated pSMAD3 nuclear accumulation coincident with elevations in the nuclear content of the C-terminal pSMAD phosphatase PPM1A (**Figure 8H**). Addition of Y-27632 prior to TGF-β1 stimulation rescued nuclear levels of PPM1 to that approximating control conditions with the corresponding characteristic decrease in nuclear pSMAD3 evident at 4 hours post-TGF-β1 addition (**Figure 8C,H**). These data suggest that ROCK regulates PPM1A levels modulating, thereby, pSMAD3 nuclear abundance. Consistent with the concept that PPM1A is a negative regulator of TGF-β1/SMAD2/3 signaling, suppression of endogenous PPM1A in VSMCs with siRNA constructs further augments TGF-β1-induced PAI-1 expression compared to identically-stimulated control siRNA transfectants(**Figure 8I**). Collectively, these findings implicate PPM1A in TGF-β1 signaling pathways in VSMCs.

## Discussion

**Figure 9:**
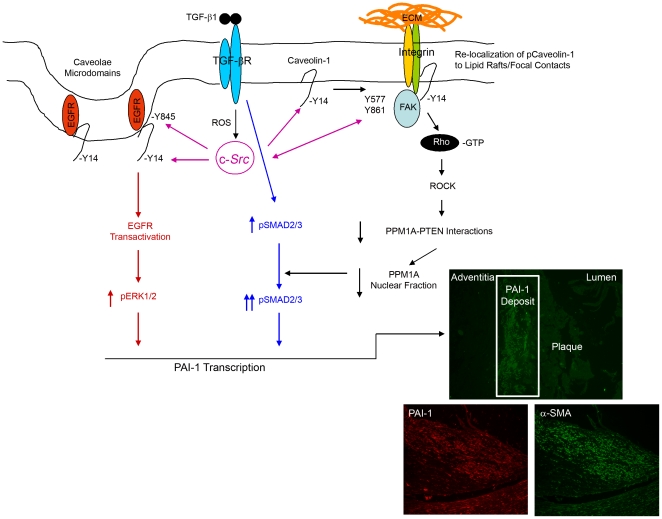
A model for TGF-β1 stimulated maintenance of SMAD3 phosphorylation and PAI-1 induction via *Src*/FAK/Caveolin-1 signaling. TGF-β1 stimulates caveolin-1^Y14^ phosphorylation in a reactive oxygen species-FAK/*c-Src* dependent manner removing repressive influences on EGFR signaling (in red) leading to EGFR transactivation (also by *c-Src*), thereby, initiating signaling events leading to the MEK-ERK pathway activation necessary for PAI-1 induction. *Src* kinase phosphorylation of caveolin-1^Y14^ also stimulates Rho-GTP loading and ROCK (an established downstream target of Rho) activation is necessary for PAI-1 induction. pCaveolin-1^Y14^-Rho-ROCK mediated signaling leads to inhibition of PTEN-PPM1A interactions resulting in a reduction of nuclear PPM1A phosphatase (black pathway), thereby, maintaining the pSMAD2/3 levels (highlighted in blue) required for PAI-1 induction by TGF-β1 (see text). PAI-1 is elevated in atherosclerotic plaques frequently colocalizing with α-smooth muscle actin-expressing cells, presumably VSMCs (insert).

VSMCs contribute to neointima formation, arteriosclerosis and vascular remodeling, particularly in the context of elevated tissue TGF-β1 and PAI-1 (**Figure 9**) [1], [11], [12], [30]. TGF-β1-induced genetic reprogramming utilizes SMAD as well as non-SMAD cascades [13], [14], [31]–[33] and while the function of SMADs as transcriptional regulators of TGF-β1 signaling is well established (e.g., [34]), how non-SMAD elements (e.g., Rho-ROCK, *Src*, FAK, caveolin-1) integrate into canonical SMAD pathways may be both cell type- and target gene-dependent. ROS generation stimulated by TGF-β1 appears to be a central element in the mobilization of the *Src*-FAK-caveolin-1-Rho-ROCK sequence leading to the maintenance of SMAD-dependent transcriptional mechanisms in VSMCs and embryonic fibroblasts. Clearly, ROS participation in ERK1/2 phosphorylation and PAI-1 gene control differs as a function of the specific stimulus (i.e., TGF-β1 vs. EGF). pp60^c-*src*^ kinase activation in response to TGF-β1, furthermore, is required for EGFR^Y845^ phosphorylation and subsequent PAI-1 gene induction via ERK1/2 dependent mechanisms in VSMCs [14], [15]. ROS-stimulated *Src* kinase activity, moreover, maintains SMAD3-dependent signaling, highlighting a central role of *Src* kinases in the regulation of both canonical (SMAD-centric) and non-canonical (e.g., EGFR-ERK/RhoA-ROCK) cascades that cooperate to attain maximal PAI-1 expression [14].

*Src* kinases are upstream effectors of both FAK and caveolin-1 activation as FAK^Y577 and Y861^ and caveolin-1^Y14^ phosphorylation upon TGF-β1 stimulation is not detected in triple-deficient SYF^−/−/−^ cells. Stable reconstitution of pp60*^c-Src^* expression in SYF-null cells rescued caveolin-1^Y14^ phosphorylation and PAI-1 induction in response to TGF-β1. Moreover, FAK also impacts caveolin-1^Y14^ site phosphorylation in the TGF-β1 signaling cascade since phospho-caveolin^Y14^ is undetectable in FAK^−/−^ cells. TGF-β1 fails to induce PAI-1 in caveolin-1^−/−^ fibroblasts while re-expression of a wild-type caveolin-1 construct in caveolin-1-deficient cells effectively rescued TGF-β1 inducibility of this serine protease inhibitor. Although gene-specific pathways downstream of caveolin-1 are only beginning to be defined, RhoA interacts with caveolin-1 in response to TGF-β1 and its activation is regulated by caveolin-1 as this response is attenuated in caveolin-1-null fibroblasts. These observations are also consistent with the requirements for fibronectin induction by TGF-β1 in mesangial cells which also involves *src*-caveolin-1-RhoA signaling [18]. Moreover, TGF-β1-stimulated PAI-1 expression in hepatocytes similarly requires caveolin-1-dependent signaling and SMAD2/3 activity [25].

Negative regulators of SMAD signaling also impact transcriptional and biological outcomes [16]. *Src*-deficient fibroblasts exhibit elevated expression PPM1A (a SMAD phosphatase) which accounts, at least in part, for reduced pSMAD levels as well as attenuated PAI-1 induction in response to TGF-β1, Indeed, ectopic overexpression of PPM1A in HaCaT keratinocytes suppressed, while shRNA depletion of PPM1A enhanced, PAI-1 transcription in response to TGF-β1 [19]. How PPM1A is regulated and its specific role in TGF-β1-driven pathophysiologic disorders (e.g., cardiovascular disease, tissue fibrosis, cancer progression/invasion) is not known. Long-term (4 hour) TGF-β1-stimulation reduced nuclear levels of PPM1A in VSMCs, consistent with observations that TGF-β1-induced proteosomal degradation of PPM1A involves attenuation of PPM1A-PTEN (phosphatase and tensin homologue) interactions [35]. Inhibition of Rho/ROCK signaling, moreover, prevented the TGF-β1-induced reduction in nuclear PPM1A levels, suggesting that the Rho-ROCK pathway positively mediates PPM1A degradation likely accounting for maintenance of nuclear pSMAD3 necessary for PAI-1 induction. PTEN activity and cellular location is also regulated by Rho kinases and ROCK can directly phosphorylate PTEN facilitating PTEN-Rho-ROCK interactions [36]. Complex formation may destabilize PTEN-PPM1A interactions. One model consistent with current data suggests that Rho phosphorylates PTEN causing dissociation of PTEN-PPM1A complexes resulting in PPM1A degradation, thereby, retaining SMAD transcriptional activity (**Figure 9**). PTEN knockdown, moreover, results in hyper-induction of PAI-1 expression in response to TGF-β1 [37] and PTEN deletion in fibroblasts is sufficient to induce PAI-1 and cellular senescence [38], [39]. Recent findings suggest that TGF-β1 induces a “senescence-like” growth arrest, at least in primary VSMCs, with accompanying increases in p21, PAI-1 and CTGF expression (unpublished). Current studies focus on evaluation of the role of PAI-1, induced via ROS/caveolin-1/SMAD-dependent signaling, in this response.
